# Southern Medical College Commencement

**Published:** 1889-03

**Authors:** 


### EDITORIALS AND MISCELLANEOUS.

SOUTHERN MEDICAL COLLEGE COMMENCEMENT.—The commencement exercises of the Southern Medical College appointed for the evening of the 2nd of March, will not take place until after we go to press. In our April nnmber we will publish an account of the interesting occasion. At this writing we learn that there are about 40 applicants for graduation. The examinations are going on, and this school has established a high standard. Much interest and anxiety mark the closing days of the session.

